# Ruptured pseudocyst of pancreas presenting with paraplegia: a case report

**DOI:** 10.1186/1757-1626-2-9336

**Published:** 2009-12-16

**Authors:** Soni Soumian, Natarajan Manimaran, Bruce Jones

**Affiliations:** 1 Department of Surgery, Good Hope Hospital, Sutton Coldfield, Birmingham B75 7RR, UK

## Abstract

**Introduction:**

Acute pancreatitis is recognised to cause both local and extra pancreatic systemic complications. Sequelae like pseudocyst of the pancreas can further be complicated by infection, intracystic haemorrhage and rupture with high mortality and morbidity. Extra pancreatic manifestations include alterations in blood coagulation factors and pro-thrombotic tendencies.

**Case presentation:**

We report an atypical presentation of intracystic haemorrhage and rupture of a pancreatic pseudocyst into the peritoneal cavity, presenting with acute onset paraplegia.

**Conclusion:**

Considering the serious consequences of this pathology, high index of suspicion, early identification and management of pancreatic pseudocyst is paramount in reducing morbidity and mortality.

## Introduction

Acute pancreatitis is recognised to cause both local and extra pancreatic systemic complications. Pancreatic pseudocyst is a local complication of acute pancreatitis. On rare occasions, it has proven lethal due to its progression to infection, intracystic haemorrhage or free rupture into the peritoneal cavity. Intracystic haemorrhage and haemorrhagic complications of pancreatitis have a reported 60-80% mortality rate [1]. Extra pancreatic manifestations include alterations in blood coagulation factors [2]. Microembolism has been suggested as a part of aetiological processes involved in the systemic effect of pancreatitis, but the clear understanding of the pathway is yet to be ascertained. We report an atypical presentation of intracystic haemorrhage and free rupture of a pancreatic pseudocyst into the peritoneal cavity, presenting with acute onset paraplegia.

## Case presentation

A 45 year-old British Caucasian male, awaiting a magnetic resonance imaging (MRI) of the lumbar spine for longstanding back pain, experienced a sudden onset of severe chest pain radiating to the back and collapsed. On arrival at the scene, the paramedics found him hypothermic (34.5°C) but cardiovascular parameters were within normal limits. Electrocardiogram confirmed sinus tachycardia. His significant past history included previous hospital admission six years ago with acute gallstone pancreatitis (computerized tomography (CT) confirmed), followed by laparoscopic cholecystectomy. Following this, he continued to have intermittent pain for which he was admitted twice with slightly elevated alkaline phosphatase and amylase. As an ultrasound scan of the abdomen was normal, subsequent endoscopic retrograde cholangiopancreaticography (with sphincterotomy) was performed but did not show any residual stones in the common bile duct. He was discharged from follow up a year later after a three-month pain free period and normalised blood tests.

In the Emergency department, clinical examination revealed a soft abdomen without any tenderness, mottling of the lower abdomen and flaccid paraplegia. Absence of tenderness was probably related to the acute onset of paraplegia with sensory and motor loss below the level of 6^th ^thoracic vertebra. Initial blood tests showed haemoglobin of 15.1 g/dl (range = 12 - 18), white cell count 8.5 × 10^9^/l (range = 3.5 - 11), amylase 80 (range = 28-100) and C reactive protein <1 (range = 0 - 4). The rest of the blood tests were also within normal limits. However arterial blood gas examination revealed severe metabolic acidosis with elevated lactate levels (pH: 7.14, HCO3: 13.2 mmol/l, base excess: -15.1 mmol/l, lactate: 10.29). Chest X-ray did not show any mediastinal widening or pneumothorax.

**Figure 1:**
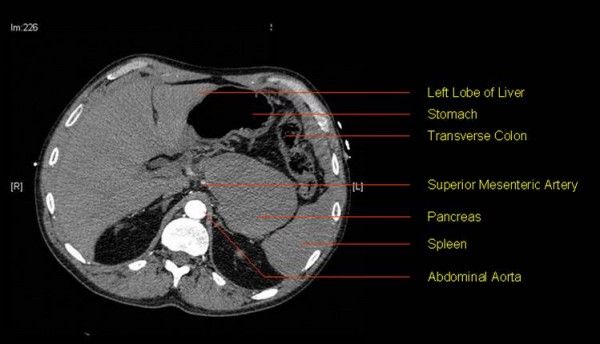
**CT abdomen showing non-enhancing lesion in the tail of pancreas**. Image in arterial phase taken before pancreas enhancement.

Based on the clinical presentation, aortic dissection had to be ruled out. Urgent contrast-enhanced CT of chest and abdomen showed a non-enhancing 7.5 cm lesion in the tail of pancreas with small amount of free fluid in the left flank and pelvis in addition to features suggesting acute pancreatitis (Figure 1). Following the scan, the patient was transferred to the intensive care unit. His condition subsequently deteriorated with onset of hypotension, acidosis, coagulation abnormalities and drop in haemoglobin levels and he died despite resuscitation.

Post-mortem showed a 9 cm pancreatic pseudocyst in the tail of pancreas with perforation in its inferior aspect and evidence of bleeding into the cyst. Three litres of blood was found in the peritoneal cavity. No clear source of bleeding was identified though the cyst wall was closely apposed to the splenic artery. No evidence of a splenic artery pseudoaneurysm was seen. Unfortunately the spinal cord was not examined and paraplegia was presumed to be due to cord ischemia due to an unexplained cause.

## Discussion

It is known that the mortality of rupture of pancreatic pseudocyst into peritoneal cavity associated with intracystic haemorrhage is high, whilst the incidence of this event is very rare. Our patient was asymptomatic when he was discharged from surgical follow up. He had no documented long-standing history of recurrent abdominal pain although he was awaiting an MRI scan for recent onset of back pain. Presentation with acute onset of paraplegia with a background of chest and back pain leads to suspicion of aortic dissection. Absence of abdominal tenderness due to sensory loss masked the abdominal signs and to add to the difficulty in diagnosis, the CT scan showed an expanded tail of pancreas suggesting pancreatitis but no extravasation of contrast or a pseudocyst.

Paraplegia associated with pancreatitis has been reported once in literature [3]. This report described a patient presenting with pancreatitis, pseudocyst formation, splenic vein thrombosis, splenic infarction and spinal cord infarction (MRI confirmed) associated with paraplegia. Our patient presented acutely with pancreatic pseudocyst rupture and haemorrhage with paraplegia (with no previous history of diagnosis of a pseudocyst). Considering the sequence of events and the rapid deterioration of the patient, no further investigation could be performed to assess the nature of spinal cord pathology.

Apart from the local complications like pseudocyst, necrosis and infection, acute pancreatitis is also known to cause systemic complications including multiorgan failure, disseminated intravascular coagulation and thrombotic tendencies. Micro embolism to the distal vasculature including fat embolism [4] has been documented in literature. The vascular effects of pancreatitis can be attributed either to its local effect on nearby vessels like the splenic vessels and coeliac axis or its promotion of systemic hypercoagulability as evidenced by the incidence of mesenteric vein, inferior vena cava [5], and spinal artery thrombosis. We acknowledge that it is difficult to demonstrate the causal relationship between paraplegia due to the ruptured pseudocyst and haemorrhage, as the spinal cord was not assessed in the postmortem evaluation. This peculiar presentation could be attributed to hypotension however on admission the patient had paraplegia with normal blood pressure and hemoglobin levels. It is also possible to speculate that an acute thromboembolic event following cardiac arrhythmias or systemic hypercoagulability resulting in either embolism to the spinal artery or spinal artery thrombosis could have been the cause of paraplegia in our patient. High index of suspicion, early identification and management of pancreatic pseudocysts are paramount in reducing the morbidity and mortality of this complication.

## Consent

No formal consent could be obtained as the patient died during the admission and reasonable efforts to obtain consent were made.

## Competing interests

The authors declare that they have no competing interests.

## Authors' contributions

SS collected the data and wrote the case report. NM and BJ contributed to the paper. All authors read and approved the final manuscript.
